# Individual and Relational Well-Being at the Start of an ART Treatment: A Focus on Partners’ Gender Differences

**DOI:** 10.3389/fpsyg.2020.02027

**Published:** 2020-09-25

**Authors:** Sara Molgora, Maria Pia Baldini, Giancarlo Tamanza, Edgardo Somigliana, Emanuela Saita

**Affiliations:** ^1^Department of Psychology, Catholic University of the Sacred Heart, Milan, Italy; ^2^IRCCS Ca’Granda Foundation Maggiore Policlinico Hospital, Milan, Italy; ^3^Catholic University of the Sacred Heart, Brescia, Italy

**Keywords:** infertile couple, assisted reproduction, gender differences, individual well-being, relational well-being, couple adjustment

## Abstract

Infertility and ART treatments represent stressful experiences for the couples, impacting on the overall psychological well-being of partners as well as on their couple adjustment. Several variables were analyzed as risk factors for infertility-related distress. The impact of these experiences has been well-documented in both women and men, reporting important gender differences. The aim of this study was to assess gender differences in individual and relational well-being in infertile couples. Gender differences for psychological and medical variables predicting psychological distress were investigated. Two hundred and thirty couples who entered an ART program at a public hospital in Milan were recruited. Each partner completed the following scales: ScreenIVF, Dyadic Adjustment Scale, and Experience in Close Relationship Questionnaire. Findings revealed several gender differences with women reporting higher levels of both anxiety and depressive symptoms, anxiety and avoidance attachment, and helplessness, but lower levels of acceptance than men. Differences emerged also in factors predicting well-being: poor support predicted anxiety in men and depression in women. Furthermore, individual well-being was predicted only for men by attachment anxiety and previous treatment. Finally, in the women subsample, couple’s adjustment was predicted by anxiety attachment, while in men predictors were helplessness and type of diagnosis. These results suggest the importance of implementing support interventions for couples which take into consideration the specific needs and fragility of each partner as well as focusing on enhancing a sense of partnership.

## Introduction

Infertility defines a wide range of conditions that impact the possibility for a couple to have a baby through natural conception (Zegers-Hochschild et al., 2017). Although total consensus on the percentage of infertile couple is lacking, it is estimated that globally 15% of couples in the fertile life stage – corresponding to almost 190 million people worldwide – have an infertility problem (Inhorn and Patrizio, 2015; Sun et al., 2019). Important differences in this percentage were found between countries (Sun et al., 2019). For example, the American National Survey of Family Growth (NSFG) data report that about 7% of married women aged 15–44 years are infertile (Somnath, 2018). Meanwhile, the prevalence of infertility among couples of reproductive age in China was found to be 25% (Zhou et al., 2018). In Italy, it is estimated that approximately 15% of couples are infertile (Fertility Europe and Eshre, 2017). The difference in these percentages can be explained by several factors, including age of partners when they try to conceive a baby naturally; indeed, fertility declines with age both in men, more gradually, and in women, with a significant decline of conception possibility after the age of 35 (Dunson et al., 2004; Pfeifer et al., 2017).

In recent years, an increasing number of infertile couples have decided to undergo assisted reproductive technology (ART) treatments to have the possibility of becoming parents, leading to an increase of babies born by means of these techniques; currently the percentage of these newborns is around 2.4%, reaching 3% among Italian newborns (European IVF-monitoring Consortium [EIM] et al., 2017; Ferraretti et al., 2017; Scaravelli et al., 2017). Specifically, considering reporting from around the world, it was calculated that, in 2013, there were 5 million babies conceived through ART, and it is estimated that at the end of this century, 157 million babies – corresponding to 1.4% of global population – will be born through ART (Faddy et al., 2018). Furthermore, it was reported that in Italy, in 2017, 78,366 couples were treated with ART techniques (Scaravelli et al., 2017).

ART includes several techniques that involve different levels of medicalization. In particular, the most widely used technique is intracytoplasmic sperm injection (ICSI, which is the direct injection of a man’s sperm into the woman’s egg) with a percentage of 46.6%, followed by frozen embryo replacement (FER, which is the thawing of frozen embryos that are replaced in the uterine cavity), and *in vitro* fertilization (IVF, wherein the man’s sperm and the woman’s egg are put in a culture dish in laboratory), accounting for, respectively, 24.7 and 18.8% of the total treatment cycles (De Geyter et al., 2018).

Infertility and ART treatments represent critical and stressful experiences for the affected couples (De Berardis et al., 2014; Koert and Daniluk, 2018). Although the nature of the association between stress and infertility is debated, data found that individuals, especially women, with infertility problems report high levels of stress, suggesting that infertility predicts (causes) stress (Rooney and Domar, 2018). Specifically, research has well recognized how these experiences, exposing partners to an unexpected crisis, can lead to negative changes in both psychological well-being and social relationships.

Considering the psychological well-being of partners, several studies found that the infertility experience has an impact on overall psychological health and the quality of life of both women and men (Schmidt, 2006; El Kissi et al., 2013; Maroufizadeh et al., 2015; Martins et al., 2016; Péloquin et al., 2018). Specifically, this condition leads to a loss or a deterioration of self-esteem as well as a negative change in one’s own identity with a risk of failure in adult identity building (Wischmann et al., 2014; Alamin et al., 2020). Furthermore, findings of previous studies reported higher levels of anxiety and depressive symptoms among infertile individuals/couples compared to fertile ones (Lakatos et al., 2017; Fallahzadeh et al., 2019). The presence of those symptoms is also related to negative cognition about infertility and with feelings of helplessness, lack of control, and lack of acceptance of the infertile condition (Patel et al., 2018).

As for relational outcomes, literature focused specifically on the impact of infertility on marital satisfaction and couple’s adjustment, with contrasting findings (Tao et al., 2012; Van Der Merwe and Greeff, 2015; Chaves et al., 2018). Indeed, while some authors found that the infertility experience leads to a decrease in couple relationship and quality (Van Der Merwe and Greeff, 2015; Gana and Jakubowska, 2016), others reported that infertility does not reduce couple satisfaction and even increases it, strengthening the communication between partners (Monga et al., 2004; Schmidt et al., 2005; Amiri et al., 2016). This difference can be explained by methodological issues as well as the role of several variables in mediating or moderating the association between infertility and marital adjustment (Ghafouri et al., 2016; Pasha et al., 2017; Greil et al., 2018).

The impact of infertility and ART has been well-documented in both women and men; in this direction, it is important to notice that important gender differences on individual and relational well-being have been reported (Bayley et al., 2009; Davidovà and Pechovà, 2014; Ying et al., 2015; Bai et al., 2019).

Specifically, women seemed to be more emotionally distressed and presented higher levels of stress, anxiety, and depressive symptoms and lower levels of self-esteem and overall quality of life than men (Berghuis and Stanton, 2002; Agostini et al., 2017; Kroemeke and Kubicka, 2018; Patel et al., 2018; Meléndez et al., 2019). Furthermore, gender differences emerged about infertility-related cognitions and, specifically, for perception of helplessness and acceptance of infertility (Patel et al., 2018); in particular, women accept ART to a greater extent than men, but men can accept childlessness more often than women (Nagórska et al., 2019). This finding suggests that women are more committed, but, at the same time, they are more distressed and emotionally concerned by their infertility problem than men, suggesting a possible explanation for the different impact of infertility diagnosis and ART treatments on male’s and female’s psychological well-being (Nagórska et al., 2019).

Significant differences between males and females also emerged for relational well-being, although findings are contrasting. For example, Peterson et al. (2011) reported higher levels of marital benefit as a positive consequence of the infertility experience among women compared with their partners. A similar result was found by Greil et al. (2018), who reported that women were more satisfied with their relationship than men, when neither partner self-identified as having a fertility problem. On the contrary, Lee and Sun (2000) found that women were less satisfied than their husbands with the couple relationship. And again, Yazdani et al. (2016) did not find any difference in marital satisfaction and adjustment between wives and husbands. These contrasting results can be partially explained considering the specific dimensions of couple relationships investigated in those studies. For example, considering the sexual dimension of the relationship specifically, experiencing sexual coercion during intercourse for conception was associated with psychological distress and poor relationship adjustment only for men, representing a threat to their masculine identity (Peterson and Buday, 2020). In any case, gender-related consequences of infertility and ART on couple’s relationships needs to be further investigated.

Several variables (e.g., socio-demographic factors, personality characteristics, fertility-related characteristics, social variables, etc.) were analyzed as risk factors in leading to infertility-related distress. For example, age was associated with sexual functioning in infertile couples, with individuals younger than 40 years old reporting a higher sexual impact than older subjects (Winkelman et al., 2016).

Furthermore, as to personality traits, neuroticism and self-criticism as well as dysfunctional romantic attachment styles (anxiety and avoidance) were found to be positively associated with global emotional infertility stress in both women and men (Lowyck et al., 2009; Donarelli et al., 2012; Rockliff et al., 2014; Theodoridou et al., 2016; Molgora et al., 2019a). With reference to fertility-related dimensions, duration of infertility, frequencies of treatments, and infertility diagnosis (that is, the cause of infertility, which can be male factor, female factor, mixed factor, or idiopathic/unexplained factor) were found to be associated with different levels of distress (Patel et al., 2016, 2018; Ma et al., 2018). In particular, Patel et al. (2018) found that distress increased after previous treatments’ failure. And again, unexplained infertility was found to be associated with the highest sexual impact (Winkelman et al., 2016). A similar result was reported by Warchol-Biedermann (2019), who found that participants with a mixed or idiopathic factor of infertility reported higher levels of distress.

Another variable that has been examined in relation to infertility and medical treatments was social support. Findings revealed that receiving and providing support had positive effects in both men and women (Kroemeke and Kubicka, 2018). Moreover, it seems that partner’s support has a protective role in facing infertility-related stress, whereas support provided by people outside the dyad has an adverse effect (Casu et al., 2019).

Gender differences also emerged for variables predicting psychological distress (Zurlo et al., 2019). For example, Donarelli et al. (2016) found that women’s distress was predicted by their own and their partner’s attachment avoidance, whereas men’s distress was predicted by their partner’s attachment anxiety. Furthermore, longer duration of infertility, higher frequencies of treatments, and female factor infertility were considered as risk factors for depression in women (Ma et al., 2018); on the other hand, Patel et al. (2018) found that men reported higher levels of distress when they were responsible for the couple’s inability to have a baby. Gender differences also emerged for social support: women benefited more from support, and their well-being was more dependent on perceived support (provided and received) than men (Kroemeke and Kubicka, 2018).

To face infertility and ART treatments, gender-specific coping strategies have been identified: specifically, women reported more emotion-focused coping strategies, while men preferred problem-focused coping strategies (Peterson et al., 2006). Moreover, women’s typical coping mechanisms were seeking professional support and social support, and taking responsibility, while men’s elective coping mechanisms were found to be distancing and self-control (Peterson et al., 2008; Pásztor et al., 2018). Finally, both partners spent time on tasks related to family-building before starting treatment and, in this case also, gender differences in the amount of time spent on these tasks emerged (Cusatis et al., 2019). Findings revealed that women’s mechanisms tended to be more successful – that is, were connected with lower levels of infertility-related psychological distress – compared to those of men (Shapiro, 2009; Pásztor et al., 2018). Coping strategies can be considered another type of predictive factors of individuals’ adjustment to infertility and ART techniques (Rockliff et al., 2014; Patel et al., 2018).

The aim of the present study was to assess gender differences in couples facing an ART experience, focusing on both individual (anxiety and depression) and relational (couple’s adjustment) well-being as well as on some psychological dimensions that can be considered as risk/protective factors of well-being (infertility-related cognitions of helplessness and acceptance, adult romantic attachment, social support). Specifically, according to previous studies, we assume that women reported higher levels of emotional distress (anxiety symptoms and depressive symptoms) and helplessness than men, but lower levels of acceptance. Although many studies have previously focused on gender differences within couples dealing with an infertility diagnosis and an ART treatment, the results were sometimes mixed, and findings focused only on individual or relational dimensions. This study considered both individual and interpersonal dimensions of psychological well-being and predictors of well-being in an attempt to better understand and articulate these differences.

Moreover, we aimed to investigate gender differences in predictors of psychological distress; in particular, three psychological variables (infertility-related cognitions of helplessness and acceptance, adult romantic attachment, and social support) and two medical variables (type of infertility diagnosis and previous ART treatment) were analyzed for their association with psychological well-being of both men and women.

## Methods

### Participants

Eligible participants were all couples who were starting an ART program at a public hospital in Milan. No exclusion criteria were put in place. From January 2018 to December 2018, a total of 230 couples (460 subjects) were recruited to participate in this study. The mean age of participants was 36.0 (*SD* = 3.8; range = 25–44) for women and 38.5 (*SD* = 5.5; range: 26–57) for men. 59.6% of women and 43.5% of men had a degree; 32.4% of women and 40.7% of men had a high-school diploma. 58.5% of women and 38.1% of men were white-collar workers. The mean duration of the marital relationship was 9.5 years (*SD* = 4.6).

Regarding infertility diagnosis, 35.1% were female factor, 13.7% were male factor, 9.5% were mixed factor, and 41.7% were idiopathic/unexplained factor. It should be noted that this high percentage can be explained considering that the information was not obtained from medical records, but from the self-report questionnaire that couples have completed, so it was based on their knowledge. Furthermore, 78.9% of the couples had not previously underwent an ART cycle, 72.1% of the couples were involved in IVF treatment, and 17.6% in ICSI treatment.

### Measures

Each partner completed a questionnaire that included the following scales:

*ScreenIVF* (Verhaak et al., 2010). This scale, composed of 34 items, was developed to assess the emotional condition of infertile couples before the start of treatments. In particular, the instrument assessed five different dimensions: pretreatment anxiety (10 items, 5 for state anxiety, and 5 for trait anxiety, on a 4-point Likert scale; range 10–40), pretreatment depression (7 items, on a 4-point Likert scale; range 0–21), cognitions regarding fertility problems in terms of helplessness (6 items, on a 4-point Likert scale; range 6–24), lack of acceptance (6 items, on a 4-point Likert scale; range 6–24), and lack of perceived social support (5 items, on a 4-point Likert scale; range 5–20). Patients were considered at risk when their scores on one or more of the five dimensions were above the clinical cut-off, that is, 24 or higher for anxiety, 4 or higher for depression, 14 or higher for helplessness, 11 or lower for acceptance, and 15 or lower for social support. For each risk factor the scale produces a dichotomous score: 0 if the subject scored below the cut-off value, and 1 if he/she scored above or equal to the cut-off value, for a total score ranging from 0 (no risk factors are present) to 5 (all five risk factors are present). The instrument showed good internal consistency for both men (with Cronbach’s alpha ranging from 0.65 for depression to 0.87 for acceptance) and women (with Cronbach’s alpha ranging from 0.64 for depression to 0.88 for acceptance).

*Dyadic Adjustment Scale* (DAS) (Spanier, 1976; Gentili et al., 2002). This scale measures couple’s adjustment through 32 items: 31 items are related to specific aspects of the couple’s relationship, and one item assesses overall happiness with the relationship. The higher the score, obtained by summing the 32 items, the greater is the perceived couple’s adjustment. The instrument showed very good internal consistency for both men (Cronbach’s alpha = 0.90) and women (Cronbach’s alpha = 0.89).

*Experience in Close Relationship Questionnaire* (Brennan et al., 1998; Picardi et al., 2002). This instrument measures the adult romantic attachment style through 36 items on a 7-point Likert scale. Specifically, it is composed of two different subscales, each composed of 18 items and measuring, respectively, attachment anxiety (e.g., “I worry about being abandoned”) and avoidance (e.g., “I prefer not to show a partner how I feel deep down”). The higher the score in each dimension is, obtained by summing the item (some reversed), the higher the levels of insecurity perceived with reference to these two attachment dimensions. The instrument showed good internal consistency for both the attachment anxiety subscale (Cronbach’s alpha = 0.89 for men and 0.88 for women) and the avoidance subscale (Cronbach’s alpha = 0.85 for men and 0.82 for women).

Finally, socio-demographic (age, educational level, job situation) and clinical (diagnosis, number of previous treatments, type of treatment) information was collected.

### Procedure

This project was approved by the Institutional Review Board of the Catholic University of the Sacred Heart. Data were collected at the beginning of the assisted reproductive technology procedure. In particular, both partners were recruited at the outpatient hospital while they were undergoing preliminary exams before entering treatment (e.g., hormonal stimulation). Each partner was asked to complete an on-site questionnaire independently from the other partner, after being informed about the research aim and signing the written informed consent form. Anonymity and data confidentiality were guaranteed.

### Data Analysis

Descriptive statistics were conducted for each instrument. Bivariate correlation among variables was performed. Differences between males and females were investigated with paired-samples *t*-test. Furthermore, the chi squared test was performed to compare men and women regarding their risk status for the ScreenIVF subscale. To investigate the impact of psychological variables (infertility-related cognitions, romantic attachment, and support) and medical variables (type of infertility diagnosis and previous ART treatment) on psychological well-being (anxiety and depressive symptoms, and couple’s adjustment) in both men and women, a series of multiple linear regression analyses were performed. When predictors were dichotomous, they were recoded as dummy variables (Frazier et al., 2004).

Given the heterogeneity of subgroups’ dimensions with reference to diagnosis conditions, infertility diagnosis was recoded as a dichotomous variable: one group comprising idiopathic and both partners’ diagnosis (BPD group; 51.2%) and another group comprising one partner’s (male or female) factors diagnosis (OPD group; 48.8%), assuming that there may be a difference depending on whether or not a single partner was identified as responsible for the infertility. Indeed, while contrasting findings were reported about the differentiating impact of male vs. female factor on men’s and women’s well-being, previous studies found that individuals with a mixed factor or an idiopathic/unexplained infertility showed higher levels of distress (Winkelman et al., 2016; Warchol-Biedermann, 2019).

## Results

Descriptive statistics of the measures for both women and men are reported in Table 1. In particular, we reported mean and SD for each scale and the percentage of subject at risk for the ScreenIVF subscales.

**TABLE 1:** Descriptive statistics of the scales.

	**Women**		**Men**			
	**M (S*D*)**	**% Risk**	**M (*SD*)**	**% Risk**	**T-test**	**χ2 test**
ScreenIVF-Anxiety	19.31 (5.0)	20.0	18.50 (4.8)	16.6	2.05*	6.99**
ScreenIVF-Depression	1.01 (1.5)	7.6	0.56 (1.1)	3.6	4.31***	20.57***
ScreenIVF-Support	16.55 (3.1)	40.2	16.25 (4.0)	46.8	1.04	
ScreenIVF-Helplessness	9.39 (3.2)	12.0	8.22 (3.0)	5.9	−3.57***	19.47***
ScreenIVF-Acceptance	16.48 (3.8)	7.8	17.66 (3.8)	5.0	4.55***	
ECR-Anxiety	52.95 (16.2)		47.79 (16.4)		3.63***	5.29*
ECR-Avoidance	51.78 (20.0)		44.80 (19.6)		4.82***	7.27**
DAS	127.56 (11.6)		128.83 (11.7)		−1.47	

*p < 0.05, **p < 0.01, and ***p < 0.001.

Table 2 presents the bivariate associations between variables for the two genders.

**TABLE 2:** Bivariate correlations between variables for men and women.

	**Variables**	**1**	**2**	**3**	**4**	**5**	**6**	**7**	**8**
1.	ScreenIVF-Anxiety	0.34***	0.52***	−0.37***	0.34***	−0.17*	0.39***	0.17*	−0.51***
2.	ScreenIVF-Depression	0.50***	0.36***	−0.21**	0.22***	–0.14	0.24***	0.18*	−0.24**
3.	ScreenIVF-Support	−0.34***	−0.35***	0.36***	–0.05	0.09	−0.25***	−0.30***	0.41***
4.	ScreenIVF-Helplessness	0.46***	0.48***	−0.22**	0.33***	−0.24***	0.35***	0.14	–0.18
5.	ScreenIVF-Acceptance	−0.39***	−0.31***	0.31***	−0.48***	0.27***	−0.19*	–0.09	0.09
6.	ECR-Anxiety	0.29***	0.26***	−0.19*	0.27***	−0.22**	0.36***	−31***	−0.33***
7.	ECR-Avoidance	0.97	–0.01	–0.03	0.00	−08	0.18*	0.53***	−13
8.	DAS	−0.44***	−0.35***	0.32**	–0.11	0.31***	−0.42***	−0.21*	0.68***

*p < 0.05, **p < 0.01, and ***p < 0.001. Men correlations are reported above the diagonal, women scores are reported below. On the diagonal, correlations between men and women for each variable are reported.

As reported in Table 2, several significant correlations emerged with some gender-specific patterns. In particular, in the men’s subsample, anxiety was found to be associated with all the other variables, while depression was correlated with the other variables except for acceptance. Furthermore, support was positively correlated with couple’s adjustment and negatively associated with romantic attachment, but no correlation was found with infertility-related cognitions. These cognitions were negatively associated each other; furthermore, helplessness was positively associated with the anxiety dimension of attachment, while acceptance was negatively associated with anxiety attachment. On the contrary, no correlations were found with the avoidance dimension of attachment and couple’s adjustment. Finally, the two dimensions of romantic attachment were also negatively correlated with each other, and the anxiety dimension was negatively associated with couple’s adjustment.

In the women’s subsample, both anxiety and depression as well as support were correlated with all other variables except for avoidance. Helplessness and acceptance were negatively associated, and helplessness was also positively correlated with the anxiety dimension of romantic attachment while acceptance was negatively correlated with this dimension of attachment and positively associated with couple’s adjustment. Finally, anxiety and avoidance were positively correlated with each other, and both were negatively associated with couple’s adjustment.

As reported in Table 1, paired sample *t*-test analyses revealed several statistically significant differences between partners’ well-being. In particular, women in the sample reported higher levels of both anxiety symptoms and depressive symptoms than men. In contrast, no differences were detected for couple’s adjustment. Gender differences also emerged for some variables considered as potential predictors of psychological well-being. Specifically, women reported higher levels of helplessness than men, but lower levels of acceptance than their partners; moreover, women reported higher levels of both anxiety and avoidance dimensions of romantic attachment than men. No differences were detected for the support dimension of the ScreenIVF.

Furthermore, the chi squared test revealed differences between men’s and women’s risk status for all the subscales of ScreenIVF. In particular, women were at greater risk for anxiety symptoms, depressive symptoms, helplessness, and lack of acceptance, while men were at greater risk for lack of support.

Considering the second aim, which was to analyze differences between partners in psychological and medical factors predicting their psychological (individual and relational) well-being, the multiple regression analysis revealed that, in the women’s subsample, anxiety is predicted by helplessness [*F*_(__7_, _140__)_ = 10.222; *R*^2^ = 0.350; *p* < 0.001], as reported in Table 3. On the other hand, no significant relationship was found for the other variables.

**TABLE 3:** Multiple linear regression: effect of psychological and medical variables on anxiety in women.

**Predictors**	**b**	**SE b**	**β**	***t***	***p***
ScreenIVF-Helplessness	0.675	0.130	0.436	5.203	0.000***
ScreenIVF-Acceptance	−0.104	0.109	−0.081	−0.960	0.339
ScreenIVF-Support	−0.225	0.130	−0.131	−1.736	0.085
ECR-Anxiety	0.042	0.024	−0.134	1.778	0.078
ECR-Avoidance	0.008	0.019	0.030	0.400	0.690
Infertility diagnosis	−0.178	0.714	−0.018	−0.250	0.803
Previous treatments	0.357	0.889	0.030	0.401	0.689

*p < 0.05, **p < 0.01, and ***p < 0.001.

Furthermore, as shown in Table 4, depression was predicted by helplessness and lack of support [*F*_(__7_, _143__)_ = 13.741; *R*^2^ = 0.414; *p* < 0.001].

**TABLE 4:** Multiple linear regression: effect of psychological and medical variables on depression in women.

**Predictors**	**b**	**SE b**	**β**	***t***	***p***
ScreenIVF-Helplessness	0.251	0.036	0.541	6.919	0.000***
ScreenIVF-Acceptance	0.049	0.031	0.127	1.599	0.112
ScreenIVF-Support	−0.115	0.036	−0.227	−3.194	0.002**
ECR-Anxiety	0.013	0.007	0.138	1.955	0.053
ECR-Avoidance	0.003	0.005	0.045	0.655	0.513
Infertility diagnosis	−0.258	0.200	−0.086	−1.291	0.199
Previous treatments	−0.349	0.247	−0.098	−1.416	0.159

*p < 0.05, **p < 0.01, and ***p < 0.001.

Finally, couple’s adjustment was predicted by support and the anxiety dimension of romantic attachment, in the latter case with a negative association [Table 5; *F*_(__7_, _132__)_ = 5.070; *R*^2^ = 0.295; *p* < 0.001].

**TABLE 5:** Multiple linear regression: effect of psychological and medical variables on couple’s adjustment in women.

**Predictors**	**b**	**SE b**	**β**	***t***	***p***
ScreenIVF-Helplessness	0.320	0.409	0.088	0.782	0.436
ScreenIVF-Acceptance	0.506	0.337	0.166	1.499	0.137
ScreenIVF-Support	0.901	0.390	−0.221	2.309	0.023*
ECR-Anxiety	−0.286	0.073	−0.394	−3.925	0.000***
ECR-Avoidance	−0.089	0.057	0.057	0.057	0.057
Infertility diagnosis	−0.233	2.236	−0.010	−0.104	0.917
Previous treatments	3.353	2.236	0.125	1.302	0.196

*p < 0.05, **p < 0.01, and ***p < 0.001.

In the men’s subsample, on the other hand, anxiety was predicted by helplessness, lack of support, and anxiety attachment [*F*_(__7_, _137__)_ = 9.225; *R*^2^ = 0.332; *p* < 0.001], as reported in Table 6.

**TABLE 6:** Multiple linear regression: effect of psychological and medical variables on anxiety in men.

**Predictors**	**b**	**SE b**	**β**	***t***	***p***
ScreenIVF-Helplessness	0.461	0.121	0.301	3.804	0.000***
ScreenIVF-Acceptance	−0.080	0.095	−0.063	−0.8449	0.400
ScreenIVF-Support	−0.282	0.084	−0.258	−3.336	0.001***
ECR-Anxiety	0.049	0.023	0.181	2.138	0.034*
ECR-Avoidance	0.022	0.019	0.092	1.160	0.248
Infertility diagnosis‘	0.444	0.687	0.047	0.647	0.519
Previous treatments	−1.096	0.818	−0.098	−1.341	0.182

*p < 0.05, **p < 0.01, and ***p < 0.001.

As shown in Table 7, depression was predicted by helplessness and previous treatments: those who had already undergone treatments in the past were more depressed than those who were on the first attempt [M = 0.76, *SD* = 1.43 vs. M = 0.50, *SD* = 0.98; *F*_(__7_, _141__)_ = 4.669; *R*^2^ = 0.196; *p* < 0.001].

**TABLE 7:** Multiple linear regression: effect of psychological and medical variables on depression in men.

**Predictors**	**b**	**SE b**	**β**	***t***	***p***
ScreenIVF-Helplessness	0.094	0.031	0.261	3.041	0.003**
ScreenIVF-Acceptance	−0.002	0.024	−0.005	−0.065	0.949
ScreenIVF-Support	−0.033	0.022	−0.127	−1.526	0.129
ECR-Anxiety	0.007	0.006	0.102	1.122	0.264
ECR-Avoidance	0.007	0.005	0.124	1.469	0.144
Infertility diagnosis	0.143	0.175	0.064	0.817	0.415
Previous treatments	−0.413	0.209	−0.156	−1.979	0.050*

*p < 0.05, **p < 0.01, and ***p < 0.001.

To conclude, as reported in Table 8, couple’s adjustment was predicted by support and lower levels of helplessness; furthermore, the type of diagnosis was found to be significantly associated with men’s couple’s adjustment: those who had a mixed factor or an idiopathic infertility reported lower levels of couple’s adjustment than men with male or female infertility factor [*M* = 127.42, *SD* = 10.96 vs. *M* = 130.49, *SD* = 11.52; *F*_(__7_, _132__)_ = 3.963; *R*^2^ = 0.270; *p* = 001].

**TABLE 8:** Multiple linear regression: effect of psychological and medical variables on couple’s adjustment in men.

**Predictors**	**b**	**SE b**	**β**	***t***	***p***
ScreenIVF-Helplessness	−0.978	0.438	−0.252	−2.229	0.029*
ScreenIVF-Acceptance	0.189	0.302	0.069	0.624	0.534
ScreenIVF-Support	0.581	0.245	−0.267	2.367	0.021*
ECR-Anxiety	−0.042	0.076	−0.064	−0.549	0.585
ECR-Avoidance	−0.048	0.061	−0.087	−0.789	0.433
Infertility diagnosis	4.799	2.241	−0.218	2.141	0.036*
Previous treatments	−2.255	2.704	−0.086	−0.834	0.407

*p < 0.05, **p < 0.01, and ***p < 0.001.

## Discussion

Many couples worldwide have to face a diagnosis of infertility and, subsequently, then undergo medical treatments to become parents, although differences in the percentages among countries have been reported (Inhorn and Patrizio, 2015; Sun et al., 2019). As pointed out in the Introduction, infertility and ART treatments represent critical and potentially stressful experiences, which can lead to individual and relational distress for both partners (De Berardis et al., 2014; Koert and Daniluk, 2018). However, literature has highlighted gender differences in coping with these experiences and in their impact on individual and relational well-being (Bayley et al., 2009; Davidovà and Pechovà, 2014; Ying et al., 2015; Bai et al., 2019). Given that several aspects could be the cause of these differences, the aim of the present study was to assess gender differences in couples undergoing medical treatment for conception, focusing on both partners’ individual and relational well-being as well as on psychological factors that could be considered as risk/protective factors for well-being. Moreover, we investigated differences between men and women in psychological and medical variables predicting personal well-being.

As hypothesized, findings revealed several gender differences both in partners’ psychological well-being and in some psychological dimensions related to well-being. Furthermore, gender differences were found for the patterns of association among the investigated variables. In particular, regarding individual well-being, women reported higher levels of both anxiety symptoms and depressive symptoms than men, and were at greater risk of belonging to the clinical group (that is, with scores above the cut-off) for anxiety and depression, confirming findings of previous studies suggesting that women are generally more emotionally distressed than men and presented an overall lower quality of life when they undergo ART treatments (Berghuis and Stanton, 2002; Agostini et al., 2017; Kroemeke and Kubicka, 2018; Patel et al., 2018; Meléndez et al., 2019). On the other hand, no difference was found between husbands and wives for couple’s adjustment. This result is in line with a previous study (Yazdani et al., 2016) reporting the absence of any difference in marital adjustment between partners. Indeed, although other studies (Lee and Sun, 2000; Peterson et al., 2011; Greil et al., 2018) found gender differences within couples for relational well-being, it should be noted that these differences are in opposite directions, suggesting that they are not so clear-cut.

Furthermore, as expected, women presented higher levels of helplessness and lower levels of acceptance than men and were at greater risk of being above the clinical cut-off score for negative infertility-related cognitions. This finding is in line with previous studies that found greater acceptance of the condition of infertility and childlessness in men, although women reported accepting treatments to a greater extent than men, showing more commitment and higher involvement than their partners (Patel et al., 2018; Nagórska et al., 2019). We can speculate that men and women have different reasons for having a child, and motherhood and maternal identity development are very important for a woman, explaining her lower level of acceptance (van Balen and Trimbios-Kempoer, 1995). And again, women reported higher levels of both anxiety and avoidance dimensions of romantic attachment than men, partially confirming findings of previous studies that found gender differences in adult romantic attachment. In particular, the meta-analysis by Del Giudice (2011) reported males having lower levels of anxiety than females but higher levels of avoidance, although important differences among the involved studies emerged. In this direction, for example, another study (Schmitt, 2003) reported that men were not significantly more avoidant than women across all culture. Finally, although no difference between men and women was found for support, a higher percentage of men were at greater risk of perceiving lack of support, partially confirming findings of another study that found lower levels of benefit from support among men (Kroemeke and Kubicka, 2018; Casu et al., 2019).

Gender differences also emerged for factors predicting individual and relational well-being. In particular, although both women’s and men’s anxiety and depressive symptoms were predicted by helplessness, confirming the central role of infertility-related cognitions for adjustment to infertility (Patel et al., 2018), poor support predicted only anxiety in men and only depression in women, suggesting a gender-specific pattern for support impact on individuals’ mental health (Kroemeke and Kubicka, 2018). Furthermore, individual well-being was also predicted only for men by attachment anxiety and having or not having had a previous treatment; specifically, attachment anxiety was associated with anxiety symptoms, confirming findings of a previous study that found a relationship between attachment anxiety and infertility-related stress in men (Donarelli et al., 2016), while the factor related to previous treatments predicted depressive symptoms, confirming findings of other studies that found how the failure of previous treatments increased distress (Patel et al., 2018).

As for relational well-being, support turned out to predict couple’s adjustment both in men and women, confirming the above-mentioned protective role of support for partners’ well-being, both individual and relational (Kroemeke and Kubicka, 2018). However, differences between men and women also emerged for predictors of relational well-being. Specifically, in women, couple’s adjustment was also predicted by anxiety attachment, while in men other predictors of couple’s adjustment were helplessness and type of diagnosis. In particular, husbands who had a mixed or idiopathic infertility factor reported lower levels of marital adjustment compared with other men, confirming results of other studies which underlined the role of diagnosis type in moderating the impact of infertility on individual and interpersonal well-being (Winkelman et al., 2016; Warchol-Biedermann, 2019).

This research has several limitations. First, it is a cross-sectional study that assesses partners’ well-being only at the beginning of ART treatment. This is a specific moment for the couple, because a partner’s desire to become parents is still possible; thus, although medical treatments represent a distant and uncertain outcome, starting down this path gives partners new hope of being able to fulfill their desire for parenting (Koert and Daniluk, 2018). Future studies should include different assessment points in order to better understand the trajectories of adjustment not only to an infertility diagnosis but also to medical treatment. In particular, it could be interesting to assess partners’ psychological well-being at the end of treatment, considering the role of successful vs. unsuccessful treatment on their mental health. Moreover, information about partner’s psychological well-being could be connected with the outcome of the medical treatment. Indeed, some studies underlined the impact of emotional reactions in achieving pregnancy after ART treatments, reporting an association between stress and the failure of treatment (Zhou et al., 2019; Gabnay-Nagy et al., 2020).

Second, in the present study only self-reporting instruments were administrated. Although these measures offer several advantages, it could be useful for a deeper understanding of the infertility experience to also have qualitative measures (e.g., an interview) that allow one to capture the nature of an individual’s experience. Third, some potentially important variables (e.g., the presence of other children) were not investigated; thus, future research should include these variables in order to better capture their role in shaping the experience of infertility and medical treatments and to understand the complexity of these experiences. Moreover, in this direction, it would be interesting to differentiate the impact of male factor and female factor infertility, according to the partner’s gender. Fourth, although we have investigated predictors of distress both in women and men, finding important gender differences, these differences were not explored with moderations. Thus, future studies should introduce moderation models in order to assess whether predictors have a different impact on individual and relational distress according to gender. Finally, it could be important for further investigations to carry out dyadic analysis in order to better understand the impact of treatment on the couple itself, beyond gender-related differences between partners (e.g., reciprocal influences).

Despite these limitations, the present results highlight important differences in men’s and women’s adjustment after a diagnosis of infertility. In particular, although there were some common predictive factors of individual and relational well-being across partners (that is, helplessness, support, and attachment anxiety), differences emerged with reference to patterns of prediction. Furthermore, medical factors turned out to predict individual and relational well-being only for men. This finding is partially congruent with previous studies that reported that the distress experienced by the partners does not depend on ART techniques (Lowyck et al., 2009; Sina et al., 2010; Van Der Merwe and Greeff, 2015). It is possible to surmise that the higher commitment of women in ART treatments and their greater acceptance of these treatments gives a lower weight to the medical variables directly related to the treatments, compared to other variables (Nagórska et al., 2019). These findings suggest the importance of implementing support interventions for couples which take into consideration the specific needs and fragility of each partner (Kroemeke and Kubicka, 2018) as well as focusing on maintaining and enhancing a sense of partnership. Indeed, it appears that the couple in this specific moment (i.e., the beginning of an ART treatment), can function as an important resource for partners’ distress and fatigue (Molgora et al., 2019b). Psychological support should be offered to all infertile couples, given that most couples desired to be supported but only about one in two couples actively seeks and asks for support, which could also be because of a lack of information about support services (Read et al., 2014). Indeed, these results emphasize the usefulness of maintaining and improving support between partners for strengthening their abilities to cope with the infertility experience and to reduce their negative effects and cognitions (Wischmann, 2008).

## Data Availability Statement

The raw data supporting the conclusions of this article will be made available by the authors, without undue reservation, to any qualified researcher.

## Ethics Statement

The studies involving human participants were reviewed and approved by the Institutional Review Board of the Catholic University of the Sacred Heart. The patients/participants provided their written informed consent to participate in this study.

## Author Contributions

SM contributed to developing the study design, to perform data analysis and to writing the entire manuscript. MB contributed to the data collection. GT contributed to writing the introduction, to perform analyses, and interpreting the results. EdS contributed to developing the study design and to supervising the research project. EmS contributed to developing the study design, to supervising the research project, and to writing the discussion section. All authors contributed to the article and approved the submitted version.

## Conflict of Interest

The authors declare that the research was conducted in the absence of any commercial or financial relationships that could be construed as a potential conflict of interest.
